# Disrupted thyroid hormone–mitochondrial coupling in diabetic cardiomyopathy: a candidate upstream amplifier of metabolic and redox maladaptation

**DOI:** 10.3389/fendo.2026.1987603

**Published:** 2026-09-22

**Authors:** Yebin Xia, Wenjie Zhang, Jinjian Guo

**Affiliations:** Department of Cardiology, The Second Affiliated Hospital of Fujian University of Traditional Chinese Medicine, Fuzhou, Fujian, China

**Keywords:** diabetic cardiomyopathy, DIO3, metabolic flexibility, mitochondria, mitochondrial quality control, redox signaling, thyroid hormone

## Abstract

Diabetic cardiomyopathy (DCM) is characterized by mitochondrial metabolic inflexibility, redox imbalance and defective organelle quality control, yet the endocrine program coordinating these abnormalities remains incompletely defined. Thyroid hormone (TH) is a major regulator of cardiac energy metabolism and mitochondrial adaptation. In experimental diabetes, myocardial triiodothyronine (T3) can decline despite preserved circulating thyroid hormone concentrations, accompanied by type 3 iodothyronine deiodinase (DIO3) induction and altered TH transport. We propose that this local defect disrupts thyroid hormone-mitochondrial coupling and may thereby constrain adaptation of substrate oxidation, oxidative phosphorylation, redox defense and organelle turnover. Preclinical diabetic-heart studies demonstrate local TH dysregulation and show that appropriately dosed T3 treatment can reverse selected structural and functional abnormalities. Complementary cardiac studies identify mitochondrial biogenesis, reactive oxygen species control, mitophagy and calcium handling as plausible downstream effectors. Together, these findings support impaired local TH signaling as an underrecognized candidate upstream mechanism linking the diabetic endocrine environment to mitochondrial maladaptation. A tissue-specific therapeutic-window model may reconcile the adverse consequences of both deficient and excessive TH signaling while providing a framework for biomarker development, mechanistic testing and precision intervention in DCM.

## 1 Introduction

Diabetic cardiomyopathy (DCM) describes myocardial abnormalities caused by diabetes that are not adequately explained by coronary, valvular or hypertensive disease. Mitochondrial substrate overload, impaired metabolic flexibility, inefficient oxidative phosphorylation (OXPHOS) and reactive oxygen species accumulation are central to this phenotype. Defective mitochondrial quality control (MQC) further promotes hypertrophy, fibrosis, cell death and diastolic dysfunction (1–3). These processes are usually framed as direct consequences of hyperglycemia, lipid excess and insulin resistance. That account overlooks an endocrine layer that continuously adjusts mitochondrial behavior.

Thyroid hormone (TH) is a strong candidate for this regulatory layer. Triiodothyronine (T3) controls cardiac contractile phenotype, substrate use, mitochondrial biogenesis and respiratory capacity through nuclear and extranuclear mechanisms. Previous reviews have addressed TH-dependent fetal reprogramming in diabetes, glucose metabolism in insulin-resistant cardiomyocytes and the broader mitochondrial actions of TH (4–6). The remaining task is to connect these fields through a disease-specific model of DCM.

We define thyroid hormone–mitochondrial coupling as coordination between local TH availability, receptor signaling and mitochondrial adaptation. This adaptation includes substrate selection, bioenergetics, redox control and organelle turnover. Our central view is that diabetes disrupts this coupling within the heart and shifts mitochondrial adaptation outside its physiological range. Targeted searches of PubMed and OpenAlex were conducted and last updated on 02 August 2026, using combinations of “diabetic cardiomyopathy” or “diabetic heart” with “thyroid hormone”, “T3”, “DIO3”, “mitochondria”, “oxidative phosphorylation”, “redox”, “mitophagy”, “mitochondrial dynamics” and “calcium handling”. Evidence was classified as direct diabetic-heart evidence when studies used diabetic cardiac models or diabetes-exposed human cardiomyocytes or cardiac organoids to examine local TH availability or signaling together with cardiac or mitochondrial outcomes; studies conducted in non-diabetic cardiac-stress models, or DCM studies that characterized mitochondrial pathways without directly testing TH signaling, were treated as complementary mechanistic evidence. This evidence architecture supports a coherent and testable model in which local cardiac TH insufficiency acts as a candidate upstream amplifier of the metabolic and redox stress that drives DCM. The proposed framework is summarized in **Figure 1**.

**Figure 1:**
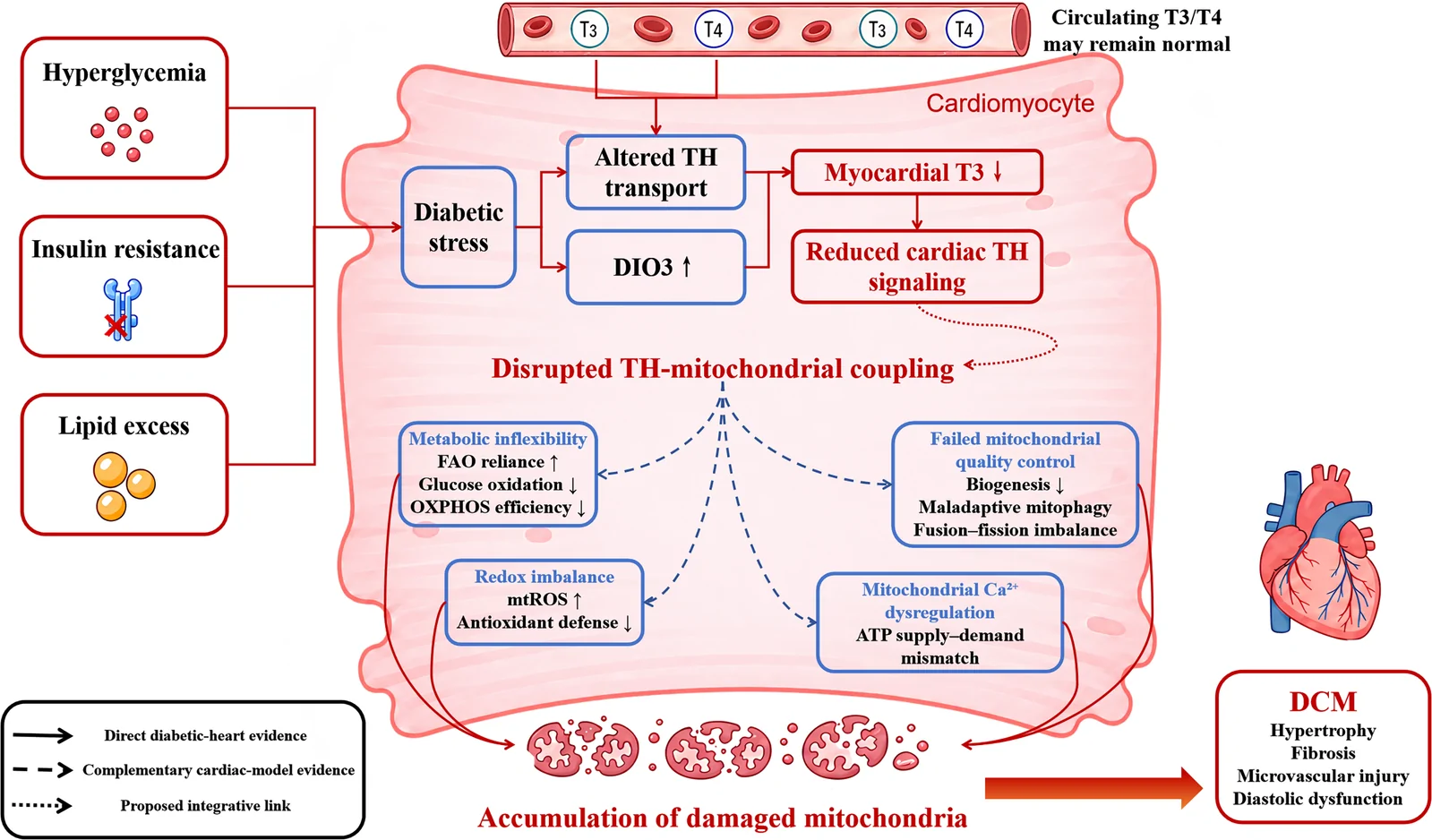
Proposed model of disrupted thyroid hormone–mitochondrial coupling in diabetic cardiomyopathy. Diabetic stress, including hyperglycemia, insulin resistance and lipid excess, may disturb local cardiac thyroid hormone (TH) homeostasis through altered TH transport and type 3 iodothyronine deiodinase (DIO3) induction. Consequently, myocardial triiodothyronine (T3) availability and cardiac TH signaling may decline even when circulating T3 and thyroxine (T4) concentrations remain within the normal range. We propose that reduced local TH signaling disrupts TH–mitochondrial coupling and thereby constrains mitochondrial adaptation. Potential downstream consequences include metabolic inflexibility with greater reliance on fatty acid oxidation, reduced glucose oxidation and impaired oxidative phosphorylation efficiency, redox imbalance with increased mitochondrial reactive oxygen species and weakened antioxidant defense, defective mitochondrial quality control involving impaired biogenesis, maladaptive mitophagy and fusion–fission imbalance, and dysregulated mitochondrial Ca²^+^ handling with ATP supply–demand mismatch. These abnormalities may promote the accumulation of damaged mitochondria and amplify established pathological features of diabetic cardiomyopathy, including hypertrophy, fibrosis, microvascular injury and diastolic dysfunction. Solid arrows indicate relationships supported directly by diabetic-heart studies, dashed arrows indicate mechanisms supported primarily by complementary cardiac models, and dotted arrows denote proposed integrative links that remain to be directly validated in DCM. DCM, diabetic cardiomyopathy; DIO3, type 3 iodothyronine deiodinase; FAO, fatty acid oxidation; mtROS, mitochondrial reactive oxygen species; OXPHOS, oxidative phosphorylation; T3, triiodothyronine; T4, thyroxine; TH, thyroid hormone.

## 2 Diabetes disrupts local cardiac thyroid hormone homeostasis

Streptozotocin-induced diabetes rapidly reduces myocardial T3 content and cardiac deiodinase activity, with incomplete normalization after insulin treatment (7). Chronic experimental diabetes provides stronger evidence for a tissue-specific defect. Myocardial T3 and thyroxine (T4) fell by approximately 39% and 17%, respectively, despite normal circulating TH concentrations. These hearts also showed increased type 3 iodothyronine deiodinase (DIO3), altered TH transporter expression and fetal-gene reactivation. The endocrine changes coincided with coronary microvascular rarefaction and impaired cardiac function. Low-dose T3 restored myocardial hormone content and attenuated these abnormalities without inducing overt hyperthyroidism (8). This study establishes a key principle: systemic euthyroidism does not guarantee cardiac euthyroidism.

Recent work extended this principle across diabetic rats, high-glucose-exposed human cardiomyocytes and human cardiac organoids. T3 treatment opposed dedifferentiation, pathological growth and cytoskeletal disorganization, while reducing mitochondrial swelling and cristae disruption (9). Diabetes-associated dysregulation of the miR-208a/myosin network further supports a fetal-like program linked to impaired TH responsiveness (10). Together, these findings identify local TH insufficiency as an active component of diabetic myocardial remodeling rather than a passive reflection of systemic illness.

Cardiac stress models clarify how this local defect may be established. DIO3 is induced during pathological hypertrophy and heart failure, thereby restricting intracellular T3 action (11). Conversely, complete myocardial DIO3 loss causes excessive local TH signaling and restrictive cardiomyopathy (12). DIO3 therefore operates as a rheostat whose sustained activation or complete absence can move the myocardium outside an adaptive TH range. A FoxO1-DIO2 pathway also regulates cardiomyocyte TH metabolism and growth (13). In human cardiomyocytes, DIO2 manipulation alters mitochondrial function, morphology and the mitochondrial unfolded protein response (14). Local cardiac TH status is therefore governed by deiodinases, transporters and receptor availability, not by serum T3 alone.

Complementary evidence comes from non-diabetic dilated cardiomyopathy. In cardiomyopathic hamsters with subclinical hypothyroidism, Khalife et al. showed that two months of TH treatment normalized resting and maximal coronary blood flow, prevented further cardiomyocyte loss and attenuated progressive left ventricular dysfunction (15). Alongside the preservation of coronary microvasculature in experimental diabetes (8), these findings support a vascular component of TH-dependent cardiac protection that could help sustain myocardial oxygen and substrate delivery.

## 3 Mitochondrial consequences of impaired thyroid hormone signaling

### 3.1 Metabolic flexibility and bioenergetics

The healthy heart switches among fatty acids, glucose, lactate and ketones according to substrate availability and workload. Diabetes narrows this flexibility and increases dependence on fatty acid oxidation (FAO). Incomplete lipid oxidation, acylcarnitine accumulation and respiratory inefficiency then reinforce myocardial stress (1–3). TH signaling regulates several nodes within this network. In insulin-resistant OLETF rats, exogenous T4 increased cardiac GLUT4 translocation (16). In post-infarct diabetic myocardium, T3-associated functional recovery coincided with AMPK and Akt/mTOR activation (17). These findings support an interaction between TH responsiveness, nutrient sensing and cardiac substrate handling under insulin-resistant or metabolically stressed conditions.

TH also promotes mitochondrial biogenesis through PGC-1α, nuclear respiratory factors and mitochondrial transcription factor A (18, 19), while influencing respiratory protein expression, mitochondrial abundance and organelle turnover. In infarcted myocardium, T3 reduced long-chain acylcarnitine accumulation while preserving mitochondrial structure (20). These TH-sensitive processes overlap mechanistically with the lipid overload and inefficient oxidation that characterize DCM. Loss of thyroid control over these pathways therefore provides a plausible route through which local TH insufficiency could exacerbate energetic maladaptation in DCM.

### 3.2 Redox adaptation and the TH-redox paradox

Mitochondrial reactive oxygen species (mtROS) function as metabolic signals but become damaging when production exceeds buffering capacity. Catalase overexpression protects cardiomyocyte structure and function in diabetic models, demonstrating a causal contribution from oxidative stress (21). Restorative TH signaling can strengthen this redox defense. T3 reduces H2O2-induced ROS and preserves mitochondrial membrane potential through PI3K-Akt signaling (22). Antioxidant treatment can also prevent low-T3 syndrome after myocardial infarction (23). Oxidative stress and local TH metabolism may therefore form a feed-forward loop that progressively weakens mitochondrial adaptation. TH-dependent protection in ischemia-reperfusion models further supports this link by reducing mitochondrial injury and cell loss (24).

The relationship is biphasic rather than uniformly protective. Excessive or prolonged TH exposure increases oxygen consumption and electron transport, which can raise ROS production beyond antioxidant capacity (6). TH also regulates mitochondrial dynamics and mitophagy in non-diabetic hearts (25, 26). These observations support a tissue-specific optimal range of TH action. Signaling below this range weakens metabolic and antioxidant adaptation, whereas signaling above it promotes hypermetabolic oxidative stress. The TH-redox paradox thus strengthens, rather than weakens, the concept of disrupted coupling by defining its physiological boundaries.

### 3.3 Mitochondrial quality control and calcium handling

MQC integrates biogenesis, fusion-fission balance, proteostasis and mitophagy. TH postconditioning reinforces PINK1-Parkin-dependent mitophagy and preserves mitochondrial membrane potential after ischemia-reperfusion injury (25). Other cardiac studies link TH signaling to mitochondrial structure, turnover and cell survival (19, 24–26). DCM studies identify the corresponding disease nodes. Mst1 suppresses SIRT3 and Parkin-dependent mitophagy, BNP promotes OPA1-mediated fusion, and PGAM5-PHB2 signaling disrupts mitochondrial surveillance (27–29). The convergence of these findings positions defective TH-sensitive MQC as a major downstream effector of local endocrine failure.

Mitochondrial calcium provides a second interface between endocrine signaling and organelle adaptation. MICU1-mediated calcium uptake supports antioxidant responses and limits DCM (30). By contrast, acid sphingomyelinase disrupts mitochondrial calcium homeostasis and promotes DCM (31). Mitochondrial calcium uniporter ablation is also protective in type 1 diabetes (32). These apparently different outcomes reflect the dependence of calcium signaling on cell type, disease stage and mitochondrial load. Early diabetic-heart studies connect altered TH responsiveness with defective sarcoplasmic reticulum calcium transport (33–35). Local TH status can therefore shape the balance between calcium-supported energy production and calcium-driven mitochondrial injury.

## 4 A therapeutic-window model reconciles conflicting evidence

Early intervention studies did not show uniformly beneficial effects of TH replacement. TH failed to normalize sarcoplasmic reticulum calcium transport, cardiac biochemistry or mechanical performance in several diabetic rat models (33–35). T3 became more effective when combined with interventions that altered fatty acid handling or carnitine availability (36, 37). These results do not invalidate the thyroid-mitochondrial model. Instead, they show that hormone responsiveness depends on the metabolic environment, treatment timing, dose, diabetes duration and residual myocardial TH signaling.

The same logic applies to DIO3. Sustained DIO3 induction can reduce local T3 and reinforce fetal-like remodeling, whereas complete DIO3 loss exposes the myocardium to excessive TH action (12). Plasma and left ventricular TH concentrations can also diverge across compensated and decompensated cardiac stress (38). We therefore propose a therapeutic-window model centered on restoration of local cardiac signaling, not empirical elevation of circulating T3. The optimal window will vary with disease stage, sex, diabetes phenotype, cell type and mitochondrial state.

This model converts apparently conflicting studies into testable predictions. TH-directed therapy should work best in hearts with documented local hormone insufficiency and preserved capacity for mitochondrial recovery. It may fail when substrate overload remains uncorrected or when irreversible organelle injury dominates. It may also become harmful when treatment drives tissue signaling above the adaptive range. These predictions make local hormone measurements and mitochondrial phenotyping essential components of future intervention studies.

## 5 Translational opportunities and complementary interventions

Translation should begin with integrated endocrine and cardiac phenotyping. In rats with hypothyroidism or post-infarction heart failure, T3 treatment reduced circulating BNP and myocardial BNP expression alongside recovery of TH-responsive gene expression and cardiac function (39). Clinical evidence likewise links lower FT3 to higher BNP (40), while low-normal FT3 is associated with adverse outcomes in euthyroid patients with heart failure receiving cardiac resynchronization therapy (41). A lower FT3/FT4 ratio, an indirect index of peripheral T4-to-T3 conversion, is also associated with increased all-cause and cardiovascular mortality in heart failure (42). Together, these findings support joint interpretation of circulating T3, the FT3/FT4 ratio and BNP to identify candidate endocrine–cardiac phenotypes that may be missed by binary reference-range classifications.

Serial assessment of this panel offers a testable approach to patient stratification and response monitoring in trials of TH-directed treatment for DCM. Establishing its ability to identify myocardial TH insufficiency and guide treatment requires prospective validation, accounting for thyroid status, renal function and cardiac loading. Mechanistic studies should pair myocardial TH content, deiodinase activity and receptor signaling with respirometry, substrate flux, mtROS and mitophagy. Human induced pluripotent stem cell-derived cardiomyocytes and multicellular organoids can provide complementary platforms for linking TH signaling to mitochondrial responses.

Thyroid-directed strategies should also be tested alongside cardiometabolic and lifestyle interventions. Exercise protects against DCM through an endocrine FGF21-SIRT3 pathway that preserves mitochondrial integrity (43). Mitochondrial STING regulates glycolytic reprogramming in DCM and provides another relevant stress node (44). These pathways illustrate how endocrine and innate stress signals converge on mitochondrial adaptation. Factorial studies can determine whether low-dose T3, selective TH receptor modulators or DIO3-directed strategies add benefit only when local TH insufficiency is present.

Traditional Chinese medicine (TCM)-derived compounds offer complementary routes to the same mitochondrial network. Icariin improves mitochondrial function through apelin-SIRT3 signaling, while paeonol promotes OPA1-mediated fusion (45, 46). Ginsenoside Rb1 modifies calcium signaling and MFN2-associated mitochondrial responses in experimental DCM (47, 48). Recent icariin studies also implicate Nrf2-dependent antioxidant pathways and SIRT3-mediated TFAM deacetylation with inflammasome suppression (49, 50). Broader TCM syntheses likewise identify MQC as a tractable therapeutic target (51, 52). These compounds may therefore serve as downstream modulators or experimental probes of thyroid-sensitive mitochondrial nodes. Their direct effects on DIO3, myocardial T3 and TRα1 remain an important question for future work.

## 6 Discussion and research priorities

We propose local TH insufficiency as a candidate organizing endocrine lesion in DCM. This framework extends DCM beyond a purely substrate-driven model by considering how diabetes may impair a hormonal program that coordinates energy production, redox defense and organelle renewal. Preclinical diabetic-heart studies provide the critical disease anchor: myocardial T3 can fall despite preserved circulating TH concentrations, DIO3 is induced, and low-dose T3 can improve selected structural and functional abnormalities (8, 9). Complementary cardiac studies then identify mitochondrial processes through which such local TH insufficiency could influence myocardial adaptation. However, direct evidence linking local TH insufficiency to mitochondrial dysfunction within the same DCM model remains limited, and myocardial TH dysregulation has not yet been sufficiently characterized in patients with DCM.

The strength of this model lies in its integration. Metabolic inflexibility, oxidative stress, defective MQC and calcium dysregulation are usually discussed as parallel features of DCM. TH signaling provides a plausible regulatory axis connecting these processes. Reduced local TH action could therefore constrain substrate switching and mitochondrial biogenesis, weaken antioxidant adaptation, impair organelle turnover and disturb calcium-energy matching. These effects could amplify the metabolic pressure generated by hyperglycemia and lipid excess. We therefore propose disrupted thyroid hormone–mitochondrial coupling as an upstream amplifier of established DCM mechanisms rather than as a fully independent pathogenic pathway.

Five questions can now test this model directly. First, is DIO3 induction necessary and sufficient for mitochondrial dysfunction in diabetic cardiomyocytes? Second, which mitochondrial domain changes first: substrate oxidation, redox signaling, calcium handling or MQC? Third, does TRα1 mediate the dominant mitochondrial response, or do transporters, DIO2 and extranuclear pathways contribute equally? Fourth, how do sex, diabetes type, obesity and disease stage alter the therapeutic window? Fifth, can serial measurements of serum T3, the FT3/FT4 ratio and BNP identify local cardiac TH insufficiency and predict treatment response without myocardial biopsy?

Future intervention studies should adopt a minimum mechanistic standard. Mechanistic validation should link circulating biomarker profiles to myocardial TH signaling and functional mitochondrial endpoints. Experiments should report myocardial hormone content, deiodinase activity, receptor signaling and at least one functional mitochondrial endpoint. High-resolution respirometry, stable-isotope tracing, compartment-specific redox sensors and quantitative mitophagy measurements should be integrated with cardiac function. Rescue designs are especially important. Genetic or pharmacological manipulation of DIO3 or TRα1 should determine whether mitochondrial correction mediates functional recovery. This approach can move the field from association toward causal validation without diluting the central hypothesis.

## 7 Conclusion

Experimental diabetes can produce a local cardiac TH deficit that may not be reflected by circulating hormone concentrations. We propose that this local endocrine disturbance can weaken a regulatory program governing mitochondrial metabolism, redox adaptation and organelle turnover. Current evidence therefore supports disrupted thyroid hormone–mitochondrial coupling as a candidate upstream amplifier of established DCM mechanisms rather than simply a secondary consequence of myocardial stress. Its translational potential lies in identifying tissue-specific TH insufficiency and restoring signaling within a stage- and dose-dependent therapeutic window. Direct causal testing and human validation will be required to determine whether this framework can provide a new endocrine entry point for precision treatment of DCM.
